# Real-Time Dynamic 3D Shape Reconstruction with SWIR InGaAs Camera

**DOI:** 10.3390/s20020521

**Published:** 2020-01-17

**Authors:** Cheng Fei, Yanyang Ma, Shan Jiang, Junliang Liu, Baoqing Sun, Yongfu Li, Yi Gu, Xian Zhao, Jiaxiong Fang

**Affiliations:** 1 Center for Optics Research and Engineering, Shandong University, Qingdao 266237, China; 18769780795@126.com (C.F.); zhaoxian@sdu.edu.cn (X.Z.); jx@sdu.edu.cn (J.F.); 2 School of Information Science and Engineering, Shandong University, Qingdao 266237, China; 201812552@mail.sdu.edu.cn (Y.M.); shanjiang0903@163.com (S.J.); julysrain@yeah.net (J.L.); baoqing.sun@sdu.edu.cn (B.S.); 3 Key Laboratory of Infrared Imaging Materials and Devices, Shanghai Institute of Technical Physics, Chinese Academy of Sciences, Shanghai 200083, China; guyi@mail.sitp.ac.cn; 4 State Key Laboratory of Functional Materials for Informatics, Shanghai Institute of Microsystem and Information Technology, Chinese Academy of Sciences, Shanghai 200050, China

**Keywords:** real-time, dynamic, three-dimensional shape reconstruction, Fourier-transform profilometry, short-wave infrared indium gallium arsenide camera

## Abstract

In this paper, a real-time, dynamic three-dimensional (3D) shape reconstruction scheme based on the Fourier-transform profilometry (FTP) method is achieved with a short-wave infrared (SWIR) indium gallium arsenide (InGaAs) camera for monitoring applications in low illumination environments. A SWIR 3D shape reconstruction system is built for generating and acquiring the SWIR two-dimensional (2D) fringe pattern of the target. The depth information of the target is reconstructed by employing an improved FTP method, which has the advantages of high reconstruction accuracy and speed. The maximum error in depth for static 3D shape reconstruction is 1.15 mm for a plastic model with a maximum depth of 36 mm. Meanwhile, a real-time 3D shape reconstruction with a frame rate of 25 Hz can be realized by this system, which has great application prospects in real-time dynamic 3D shape reconstruction, such as low illumination monitoring. In addition, for real-time dynamic 3D shape reconstruction, without considering the edge areas, the maximum error in depth among all frames is 1.42 mm for a hemisphere with a depth of 35 mm, and the maximum error of the average of all frames in depth is 0.52 mm.

## 1. Introduction

The three-dimensional (3D) shape reconstruction technique can reconstruct actual 3D targets, which can be divided into contact type and non-contact type [1]. The optical 3D shape reconstruction technique is a kind of widespread non-contact technique using optical images as a means of detection and transmitting information. It possesses the advantages of large range, non-contact, high-speed, high precision, as well as great system flexibility [2]. Currently, the optical 3D shape reconstruction technique is widely used in the fields of industrial inspection, machine vision, biomedicine, digital museum, etc. [3,4,5,6,7]. Optical 3D shape reconstruction techniques can be classified into two categories: the passive type and the active type. The passive optical 3D shape reconstruction technique does not introduce active light source for illumination, rather, it uses computer vision to extract 3D features of targets from image shadows, textures, and contours in most cases. The active optical 3D shape reconstruction technique illuminates the target by an active light source and is achieved by spatial or temporal modulation. In recent years, the structured illumination 3D shape reconstruction technique becomes more important and is widely used, which is an active spatial modulation optical 3D shape reconstruction technique [8,9]. This kind of technique designs different types of structured light and projects the structured light onto the surface of the target in different ways. Then, the deformed structured light image modulated by the surface of the target is captured by the camera, and the image is analyzed and processed by the digital image processing technique to obtain the 3D depth information of the target [10,11].

Generally, most researchers choose visible light as the light source in the structured illumination 3D shape reconstruction. The principle of short-wave infrared (SWIR) imaging is the same as that of visible light, as the light reflected from the surface of the target is generally used [12]. Consequently, SWIR is suitable as a light source in structured illumination 3D shape reconstruction as well. However, in specific applications such as monitoring in low illumination environments, SWIR light acts better than visible light. The human eye-safe wave band is included in the SWIR wave band, therefore, SWIR light in an appropriate power does not do harm to the human body [13,14]. Based on this characteristic, SWIR light is very suitable for structured illumination 3D shape reconstruction related to human body. Other than that, SWIR light can be used to identify camouflage, and the concealability and environmental adaptability of SWIR light is superior to visible light [15,16,17].

In practice, the target is not static in many applications, to enhance the adaptability of structured illumination 3D shape reconstruction and extend its application field, sometimes applying structured illumination 3D shape reconstruction on dynamic targets is required. Hence, the research of real-time dynamic 3D shape reconstruction is really necessary [18]. In a structured illumination 3D shape reconstruction system, a high-speed camera is essential for real-time recording. For 3D shape reconstruction method, a high-speed, single-shot 3D shape reconstruction is really needed. Meanwhile, it is necessary to reconstruct multiple targets based on the fringe pattern distribution of one background image in the same scene. Fourier-transform profilometry (FTP) is a popular 3D shape reconstruction method proposed by Takeda and Mutoh in 1980s [19], which has become a powerful tool for many applications [20,21]. FTP is a single-shot reconstruction method, which means it can extract absolute phase map from a single frame of two-dimensional (2D) fringe pattern and convert the phase to depth [22,23,24]. Further, the FTP method can reach a relatively high speed due to its low computing complexity, and it is easy to carry out [25]. The characteristics of FTP method fit the needs perfectly, and hence it can be concluded that the FTP method is well suited in real-time dynamic 3D shape reconstruction for monitoring applications.

In this paper, real-time dynamic 3D shape reconstruction is realized by employing an improved FTP method for monitoring applications in low illumination environments. The improved FTP method has the advantages of reducing the error ratio during phase unwrapping process and extracting 2D background fringe pattern from the image of 2D deformed fringe pattern without capturing a new image, which indicates that the real-time dynamic 3D shape reconstruction can obtain satisfactory reconstruction results with higher speed. In addition, all 3D shape reconstruction results in this work are quantified with exact depth values. Accuracy evaluations of the system for static and real-time dynamic 3D shape reconstruction are given as well.

## 2. Methods

In this paper, an improved FTP method is applied to achieve real-time dynamic 3D shape reconstruction.

**Figure 1:**
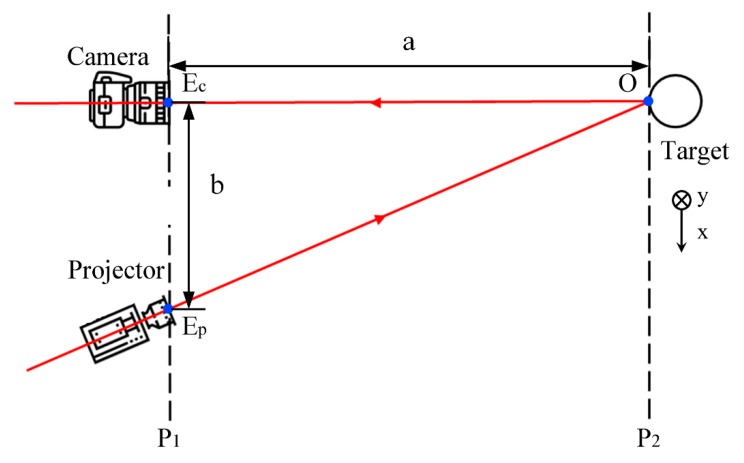
Crossed-optical-axes geometry.

For this improved FTP method, the optical geometry is the same as the conventional FTP method and can be divided into crossed-optical-axes geometry and parallel-optical-axes geometry. Here, the SWIR 3D shape reconstruction is built using the crossed-optical-axes geometry, and the geometry is shown in Figure 1.

In crossed-optical-axes geometry, the optical axes of the projector and the camera lie in the same plane and intersect a point on the surface of the target. As can be seen from Figure 1, the optical axis $E_{P}O$ of the projector crosses the other optical axis $E_{c}O$ at point O on the surface of the target. The camera and projector lens are located on the plane $P_{1}$, where point O on the surface of the target is located on a plane $P_{2}$ parallel to $P_{1}$. $E_{P}$ is the center of the pupil for the projector, and $E_{c}$ is the center of the pupil for the camera. In addition, (x,y) is the pixel coordinate of the target on the plane $P_{2}$.

Assuming that the intensity distribution of the projected fringe pattern is a cosine function along the *x*-axis, then the deformed fringe pattern modulated by the surface of the target can be given as

(1) $$\begin{array}{c} I\left(x,y\right)=a\left(x,y\right)+b\left(x,y\right)cos\left(2\pi f_{0}x+\varphi \left(x,y\right)\right)\\ =a\left(x,y\right)+c\left(x,y\right)exp\left(i2\pi f_{0}x\right)+c^{*}\left(x,y\right)exp\left(-i2\pi f_{0}x\right), \end{array}$$

where a(x,y) is the intensity distribution of background light, b(x,y) is the amplitude intensity, $f_{0}$ is the carrier frequency, and φ(x,y) contains phase information to be extracted.

Next, a 2D Fourier transform of Equation (1) is obtained through fast Fourier transform (FFT) algorithm as

(2) $$H\left(x,y\right)=A\left(f_{x},f_{y}\right)+C\left(f_{x}-f_{0},f_{y}\right)+C^{*}\left(f_{x}+f_{0},f_{y}\right),$$

where $A\left(f_{x},f_{y}\right)$, $C\left(f_{x}-f_{0},f_{y}\right)$, and $C^{*}\left(f_{x}+f_{0},f_{y}\right)$ are the Fourier spectra of a(x,y), $c\left(x,y\right)exp\left(i2\pi f_{0}x\right)$, and $c^{*}\left(x,y\right)exp\left(-i2\pi f_{0}x\right)$, respectively. $A\left(f_{x},f_{y}\right)$ is the zero frequency component corresponding to the intensity distribution of background light. $C\left(f_{x}-f_{0},f_{y}\right)$ is the fundamental frequency component corresponding to the phase information to be extracted.

For the conventional FTP method [26,27,28], a filter is designed to extract the fundamental frequency component and move it to the coordinate origin of the spectrum plane for inverse Fourier transform. Where for improved FTP method here, the fundamental frequency component is extracted for inverse Fourier transform without moving it to the coordinate origin of the spectrum plane

(3) $$d\left(x,y\right)=\frac{1}{2}a\left(x,y\right)exp\left\{i\left[\varphi \left(x,y\right)+2\pi f_{0}x\right]\right\}=\frac{1}{2}a\left(x,y\right)exp\left[i\psi \left(x,y\right)\right].$$

Here, the package phase distribution ψ(x,y) is set as the superposition of the target phase distribution φ(x,y) and the carrier frequency phase distribution $2\pi f_{0}x$, and can be expressed as

(4) $$\psi \left(x,y\right)=\varphi \left(x,y\right)+2\pi f_{0}x.$$

Then, a logarithmic operation is applied on Equation (3) and an equation is obtained as

(5) $$\log\left[d\left(x,y\right)\right]=\log\left[\frac{1}{2}a\left(x,y\right)\right]+i\psi \left(x,y\right).$$

Here, the logarithmic operation is used to extract the exponential term containing the unwrapping phase. Then, the unwrapping phase of φ(x,y) can be obtained by subtracting the unwrapping phase of $2\pi f_{0}x$ from the unwrapping phase of ψ(x,y). Here, the purpose of not separating the target phase distribution from the carrier frequency phase distribution in the improved FTP method is to reduce the error ratio during the phase unwrapping process, for the presence of the carrier frequency is beneficial to obtain the correct quality map [29]. After phase unwrapping, the depth distribution h(x,y) can be converted by the unwrapping phase φ(x,y) through

(6) $$h\left(x,y\right)=a\varphi \left(x,y\right)/\left(\varphi \left(x,y\right)-2\pi f_{0}b\right),$$

where a is the distance between plane $P_{1}$ and $P_{2}$ while b is the distance between the center of the pupils for camera and projector.

Generally, the improved FTP method in this paper has two significant advantages. On one hand, the error ratio during the phase unwrapping process can be reduced by this method, which leads to satisfactory reconstruction result. On the other hand, the 2D background fringe pattern can be extracted from the image of 2D deformed fringe pattern without capturing a new image, which leads to higher speed.

## 3. Experimental Setup and Results

**Figure 2:**
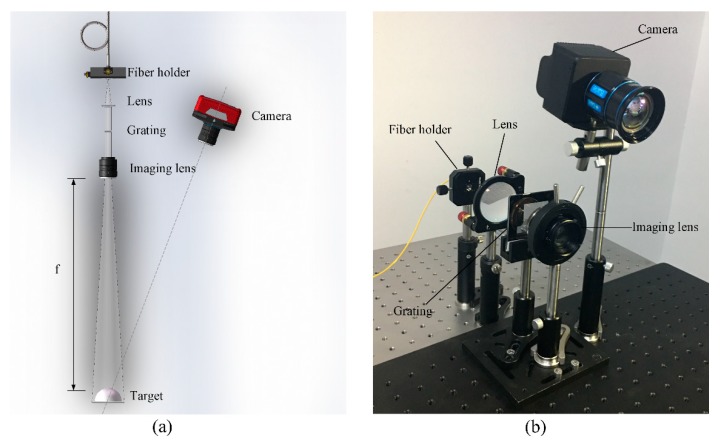
(**a**) Schematic of the three-dimensional (3D) shape reconstruction system. (**b**) Photograph of the 3D shape reconstruction system.

The schematic of the 3D shape reconstruction system is shown in Figure 2a. It consists of a fiber laser, a fiber holder, a plano-convex spherical lens, a grating, an imaging lens and a SWIR camera. The light source in the 3D shape reconstruction system is a 1550-nm fiber laser, and the power of the laser is 30 mW. The plano-convex spherical lens has a diameter of 50 mm and a focal length of 100 mm. As for the grating, it is a transmission grating with a size of 20 × 20 mm, the grating period is 4 line-pair per millimeter, and its peak transmission is achieved at 1550 nm. Further, the imaging lens of the projection part has a focal length of 35 mm. This system is a crossed-optical-axes system, and as can be seen, the optical axis of the SWIR camera and the axis of the projection system are converged at the surface of the target. The fiber of the fiber laser is fixed on a fiber holder, and then the light source here can be regarded as a point light source. Laser emitted from the fiber laser passes through the plano-convex spherical lens and becomes a collimated beam, then the beam is irradiated through the grating and the fringe pattern is projected onto the surface of the target through the imaging lens. The deformed fringe pattern on the surface of the target is obtained by the SWIR camera. The size of field of view for SWIR camera is 500 × 500 mm at the target plane, which is the measuring range of the 3D shape reconstruction system as well. Figure 2b shows the photograph of the 3D shape reconstruction system.

It should be noted that the SWIR camera used here is independently developed based on the indium gallium arsenide (InGaAs) sensor designed by Shanghai Institute of Technical Physics (SITP). The spectral range of the InGaAs sensor is from 900 to 1700 nm and the resolution of it is 640 × 512 pixels. The sensor used is a backside illumination type using planar structure and has no guard ring, the pixel pitch is 25 μm, and the spacing between neighboring pixels is 5 μm. Other than that, the SWIR camera based on the sensor has an adjustable imaging frame rate up to 400 Hz, and the imaging lens of it has a focal length of 25 mm and an F-number of 1.4.

**Figure 3:**
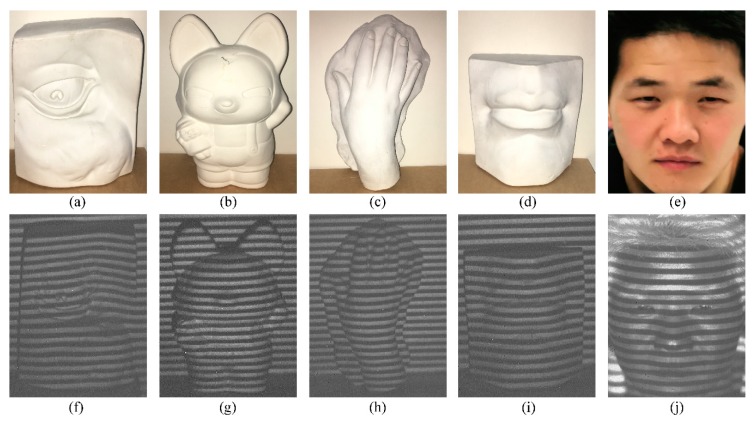
Visible images of (**a**) eye, (**b**) doll, (**c**) hand, (**d**) lip, and (**e**) real human face captured during experiment. Two-dimensional (2D) deformed fringe patterns of (**f**) eye, (**g**) doll, (**h**) hand, (**i**) lip, and (**j**) real human face.

Here, static 3D shape reconstruction on different targets is carried out first. Several plaster models of eye, doll, hand, and lip, and one real human face are chosen as targets. In order to show the characteristic of concealability, these models are captured by a visible camera during experiment, and are shown in Figure 3a–e. As can be seen from the figures, the projected fringe patterns are invisible in visible images, which indicates that it is concealed to human eyes. Here, the real human face with complex shape, is chosen as one of the targets to prove the eye-safe characteristic of SWIR, and it is more similar to the target in monitoring application. In order to obtain clear imaging results, these targets are placed at an appropriate distance from the 3D shape reconstruction system. Before putting these targets into the 3D shape reconstruction system, the background fringe pattern without target is recorded as the reference pattern, for targets can be reconstructed based on the fringe pattern of the same background image in the same scene with less errors caused by system mapping. Then, the selected targets are placed into the 3D shape reconstruction system in turn. Adjusting the focal length of the imaging lens in the projection system to ensure that the fringe patterns projected onto the surface of the targets are clear each time, and these deformed fringe patterns are precisely recorded by the SWIR camera, as shown in Figure 3f–j.

**Figure 4:**
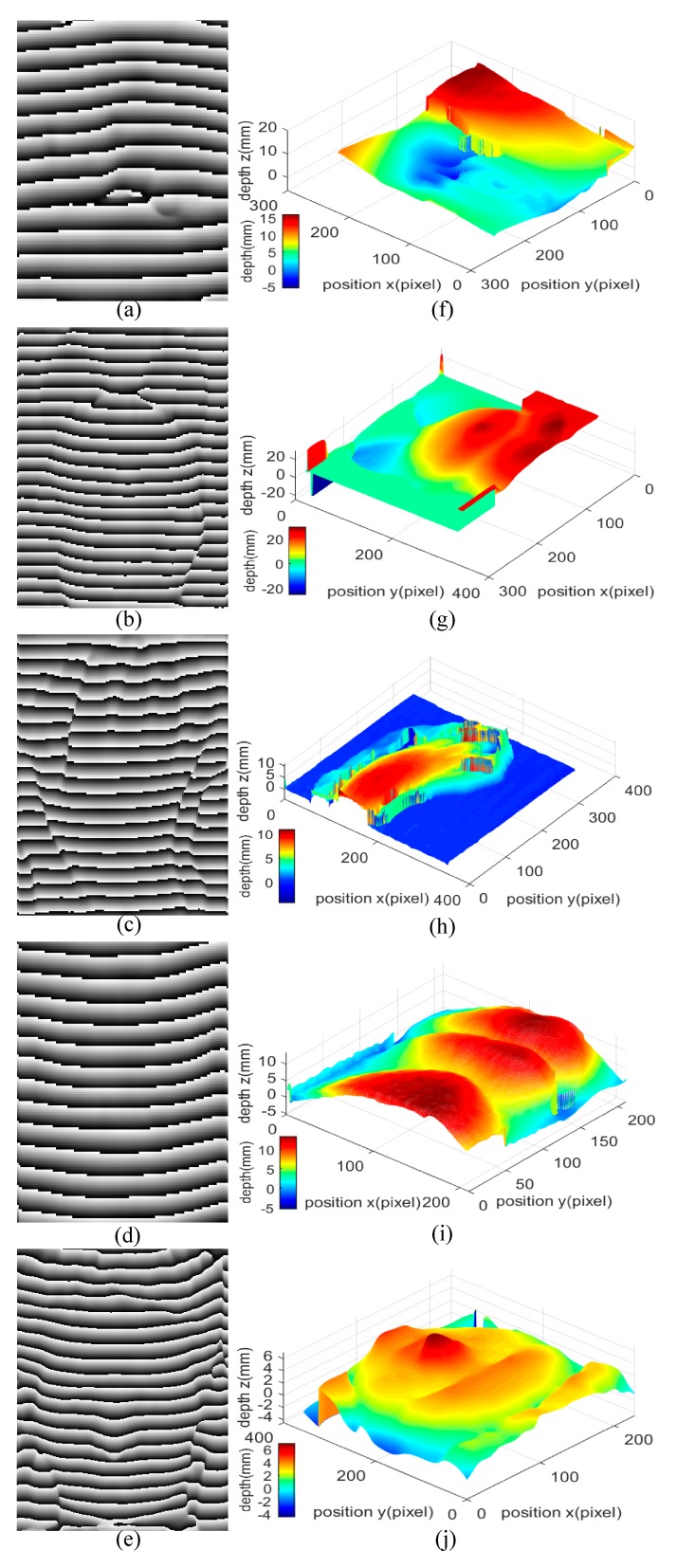
2D phase maps of (**a**) eye, (**b**) doll, (**c**) hand, (**d**) lip and (**e**) real human face. 3D reconstruction images of (**f**) eye, (**g**) doll, (**h**) hand, (**i**) lip, and (**j**) real human face.

Fringe pattern phase extraction is applied by using the improved FTP method, the 2D phase maps of the targets shown in Figure 4a–e are wrapped into [−π, π]. In order to achieve 3D shape reconstruction on these targets, phase unwrapping is performed on the previously obtained phase maps, and the phase is converted to depth at last. The errors generated from phase extraction and unwrapping reduce the accuracy and result in lower performance of the 3D shape reconstruction. Actually, the errors often come from two aspects in most cases, one is the abrupt depth changes on the surface of the target, and the other is the shadows on the surface of the target. The 3D shape reconstruction images of these targets are shown in Figure 4f–j, and all these targets are reconstructed well. As can be seen from Figure 4f, though the eye is successfully reconstructed, the result is a bit rough, with many details not revealed because of the intrinsic drawback of FTP algorithm. A rough 3D shape reconstruction result of eye is due to that the contour is complex with some abrupt depth changes on its surface, as well as the shadows on it. Where in Figure 4g,h, the 3D shape reconstruction results of doll and hand are much better than the result of eye, but still not perfect. The contours of doll and hand are as complex as the eye, but they are very smooth with fewer shadows on their surface. Therefore, the reconstruction results of them are much better than the eye. As for the lip in Figure 4i, relatively speaking, the 3D shape reconstruction result has the best performance among all results, and most of the details are satisfactorily reconstructed. Compared with those targets mentioned above, the contour of the lip is the simplest and clearest, and almost with no abrupt depth changes and shadows, which leads to a near perfect 3D shape reconstruction result. Besides, as can be seen from Figure 4j, satisfactory 3D shape reconstruction result has been achieved for the real human face, and the outlines of eye, nose, mouth, and even hair are well reconstructed with their depths close to reality. Based on the result, it is confirmed that the 3D shape reconstruction system is applicable in monitoring applications.

## 4. Accuracy Evaluation of Static 3D Shape Reconstruction

For 3D shape reconstruction, reconstruction accuracy is always the key indicator. It is necessary to make an evaluation on the reconstruction accuracy of the 3D shape reconstruction system, and the achievable depth accuracy here is used to characterize the reconstruction accuracy.

**Figure 5:**
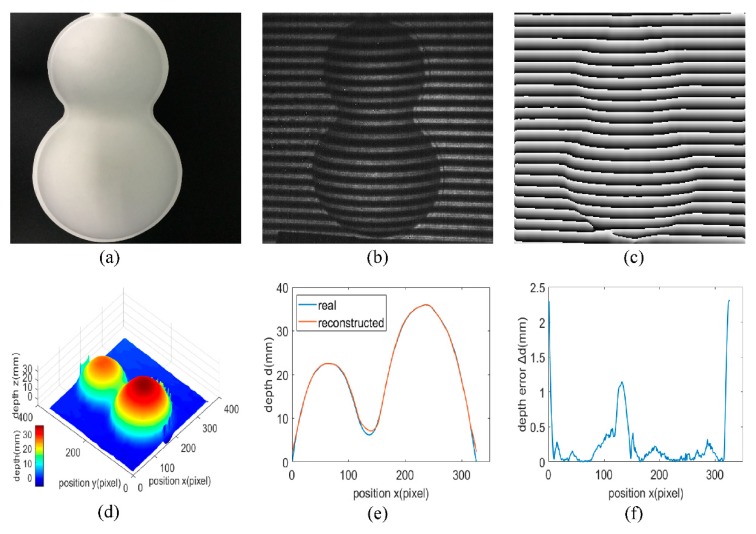
(**a**) Photograph of the plastic model. (**b**) 2D deformed fringe pattern of the plastic model. (**c**) 2D phase map of the plastic model. (**d**) 3D shape reconstruction image of the plastic model. (**e**) Reconstructed depth distribution versus true values at the symmetry axis of the effective part and (**f**) the corresponding depth error distribution.

To carry out the achievable depth accuracy test, a plastic model shaped like a gourd is chosen as the target in the 3D shape reconstruction system as shown in Figure 5a. The target is placed at a proper position from the 3D shape reconstruction system, and the focal length of the projection system and the SWIR camera are properly adjusted. Then, the fringe patterns can be clearly projected onto the plastic model surface and the deformed fringe patterns on the surface of the plastic model can be taken by the SWIR camera clearly. Figure 5b shows the 2D deformed fringe pattern image modulated by the plastic model, and the 2D phase map of the plastic model shown in Figure 5c is obtained by applying the improved FTP method on the 2D deformed fringe pattern image. Then, phase unwrapping process is carried out on the 2D phase map, and the phase is converted to depth for obtaining the 3D shape reconstruction image shown in Figure 5d. As can be seen from Figure 5a, there are abrupt depth changes at the edge of the target, which will cause great trouble in the case of 3D shape reconstruction and lead to large reconstruction errors, as proven in Figure 5d. Therefore, the reconstruction accuracy is evaluated using only the part varying evenly and gently without the edge of the target, where the maximum depth is 36 mm, and the part is regarded as effective part. For this effective part, the depth distribution of it is smooth and with no abrupt depth changes, a 3D shape reconstruction with satisfactory results can be obtained through the improved FTP method.

Figure 5e shows the reconstructed depth distribution at the symmetry axis of the effective part comparing with the true values measured by a 3D surface profilometer. The 3D surface profilometer is LJ-X8200 manufactured by Keyence with an accuracy of 1 μm and a resolution of 0.1 μm in depth, and it worked on a principle of laser triangulation. Figure 5e indicates that the reconstructed depth distribution at the symmetry axis of the effective part is not completely the same as the real depth distribution, there are depth errors between them. These depth errors are extracted as shown in Figure 5f. Since the normal of the edge of the effective part is almost perpendicular to the line of sight, the reflectance here is rather low. Therefore, it is inevitable that large depth errors exist here, and Figure 5f proves that as the maximum depth error at the edge is approximately 2.3 mm. Regardless of the depth errors at the edge of the effective part with low reflectance, the maximum depth error can be up to 1.15 mm, and it appears in the valley of the effective part, which also has a low reflectance. In areas where the depth variation is gentle and the reflectance is uniform, the maximum depth error does not exceed 0.5 mm, and hence it can be stated that the 3D shape reconstruction system here provides a high accuracy.

## 5. Real-time Dynamic 3D Shape Reconstruction

Different from the static 3D shape reconstruction, real-time dynamic 3D shape reconstruction is not aimed at fixed targets, and the targets are often in regular or irregular motion. As time goes by, the position of the target will change accordingly. Since the target may be moving at any time, in order to record 2D deformed fringe patterns real-timely, a camera with an appropriate frame rate is necessary. Here, for the SWIR camera, the adjusted frame rate can be up to 400 Hz, which is fully able to meet the requirement. In the meantime, the 3D shape reconstruction also needs to be real-time, which means it is essential to achieve 3D shape reconstruction based on only one deformed fringe pattern with high speed. In addition to this, the ability of reconstructing the target at different positions based on the fringe pattern distribution of the same background image in the same scene is also needed. Coincidently, the above requirements are precisely the advantages of the improved FTP method employed in this paper, so the improved FTP method is well suited for real-time dynamic 3D shape reconstruction.

**Figure 6:**
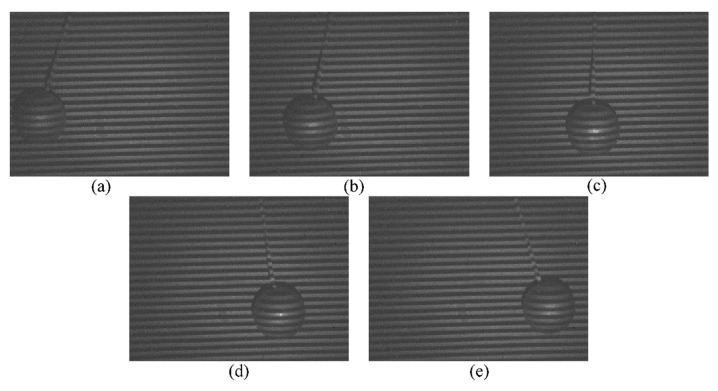
2D deformed fringe patterns of the pendulum ball at frames (**a**) 6, (**b**) 9, (**c**) 12, (**d**) 15, (**e**) 18.

In order to avoid unnecessary reconstruction errors and highlight the dynamic characteristics of the 3D shape reconstruction system, here a pendulum ball with low complexity is utilized as the target. The upper end of the rope of the pendulum ball is tied high, and the ball swings in the vertical projection direction of the fringe pattern projected area. The lens of the projection system is adjusted to ensure that the fringe patterns projected to the pendulum ball are clear, and meantime the imaging lens of SWIR camera is adjusted in order that the camera can obtain clear deformed fringe patterns. Here, considering the speed of 3D shape reconstruction, a frame rate of 25 Hz is set for the SWIR camera to record the 2D deformed fringe patterns of the whole motion of the pendulum ball. Figure 6a–e show the 2D deformed fringe patterns of the pendulum ball at frames 6, 9, 12, 15, and 18.

**Figure 7:**
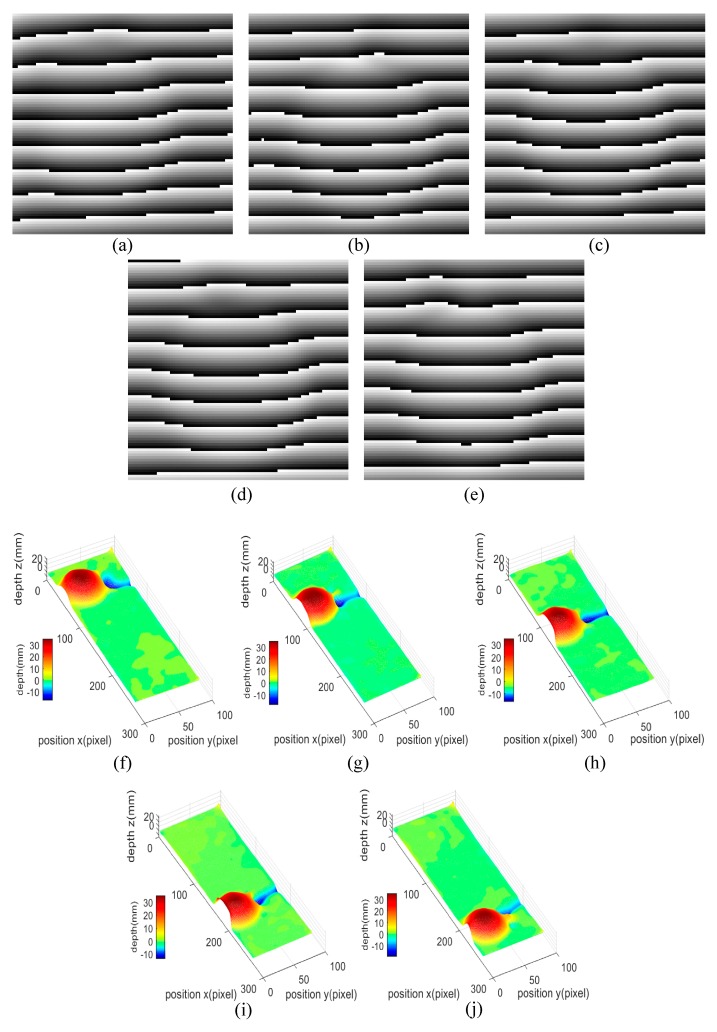
2D phase maps of the pendulum ball at frames (**a**) 6, (**b**) 9, (**c**) 12, (**d**) 15, (**e**) 18. Real-time dynamic 3D shape reconstruction results of the pendulum ball at frames (**f**) 6, (**g**) 9, (**h**) 12, (**i**) 15, (**j**) 18.

Generally, the dynamic pendulum ball is always reconstructed with small deformation, and the small deformation here is caused by the motion of the pendulum ball during the integration time of the SWIR camera. The longer the integration time is, the greater the motion amplitude of the pendulum ball per frame is, which will cause more serious deformation in 3D shape reconstruction. Therefore, shortening the integration time can solve this problem effectively and achieve a better 3D shape reconstruction result. However, shortening the integration time without adjusting the frame rate of the camera often causes discontinuity in real-time dynamic 3D shape reconstruction, so the frame rate of the camera should be increased while shortening the integration time. Besides, high frame rate and short integration time of the SWIR camera lead to a stronger light source to ensure that the camera can obtain clear fringe patterns. Hence, the integration time of the SWIR camera is set to 1 ms, and the frame rate is set to 25 Hz. Further, a 1550-nm fiber laser with a power of 30 mW is chosen as the light source. Based on the fringe patterns obtained before, real-time dynamic 3D shape reconstructions are carried out using an improved FTP method, and the phase map of the pendulum ball at these frames are shown in Figure 7a–e, with the results of the real-time dynamic 3D shape reconstruction shown in Figure 7f–j. In addition, the video of 2D deformed fringe patterns versus real-time dynamic 3D shape reconstruction results is provided as Supplementary Video S1. According to Figure 7f–j and video as well as the analysis above, it can be concluded that the 3D shape reconstruction system is fully capable of achieving real-time dynamic 3D shape reconstruction.

## 6. Accuracy Evaluation of Real-Time Dynamic 3D Shape Reconstruction

After the implementation of real-time dynamic 3D shape reconstruction, it is essential to quantify the accuracy. The pendulum ball used in this work is well suited for accuracy evaluation.

For real-time dynamic 3D shape reconstruction, different integration times and frame rates of the SWIR camera may cause different degrees of deformation of the target according to the analysis above, which lead to different errors in depth. In this section, the accuracy evaluation is applied on the results reconstructed from the image obtained by the SWIR camera with an integration time of 1 ms and a frame rate of 25 Hz. Further, in the process of accuracy evaluation, it is found that the errors also change with the motion of the pendulum ball. Consequently, accuracy evaluation is performed on each frame during the motion of the pendulum ball to find the maximum error in depth. Five consecutive frames are chosen from all frames for analyzing the changes in reconstruction results among frames. Finally, all frames are averaged to obtain the average errors in depth for analysis. Since the active light source can only illuminate one side of the pendulum ball, the result obtained by the real-time dynamic reconstruction is a hemisphere with a depth of 35 mm, then the accuracy evaluation is also carried out on the hemisphere. Here the row that crosses the center of the hemisphere is taken to evaluate the accuracy.

**Figure 8:**
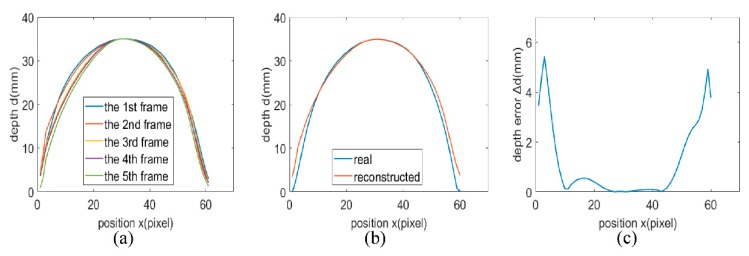
(**a**) Reconstructed depth distributions of rows in five consecutive frames. (**b**) Average reconstructed depth distribution versus true values at the row that crosses the center the hemisphere and (**c**) the corresponding depth error distribution.

The rows that cross the center of the hemisphere are extracted in all frames from the real-time dynamic 3D shape reconstruction results to compare with the real value. Then a maximum error in depth at the edge with low reflectance of 7.38 mm is found. Meanwhile, in areas with high reflectance, the maximum error is 1.42 mm. The reconstructed depth distributions of rows in the five chosen frames are shown in Figure 8a. It is obvious that the reconstructed depth distributions of rows in these frames are close to each other, which indicates that applying accuracy evaluation by averaging the reconstructed depth distributions of all rows from all frames is reasonable. Then, rows extracted from all frames are averaged, and a row which represents the average depth distribution of all rows can be obtained. Figure 8b shows the average reconstructed depth distribution versus true values at the row that crosses the center the hemisphere, and the error between them is extracted, as shown in Figure 8c. As can be seen from Figure 8c, the maximum value for average errors in depth at the edge is 5.44 mm, which is influenced by the low reflectance. Except for the edge areas, the maximum value of average errors in depth is 0.54 mm due to high reflectance and the absence of abrupt depth change. This explains why the proposed system can provide high accuracy during real-time dynamic 3D shape reconstruction.

## 7. Conclusions

In this paper, a real-time dynamic 3D shape reconstruction scheme based on the FTP method is achieved for monitoring applications in low illumination environments by using a SWIR InGaAs camera. To obtain the SWIR 2D fringe pattern, a SWIR 3D shape reconstruction system is built. An improved FTP method is applied for realizing the 3D shape reconstruction. Static 3D shape reconstruction is carried out on several models. After that, the achievable accuracy of the 3D shape reconstruction system is evaluated by using a plastic model as the target. Then, a real-time dynamic 3D shape reconstruction on a pendulum ball with a frame rate of 25 Hz are realized, and the achievable accuracy is evaluated. The SWIR 3D shape reconstruction mentioned in this paper can obtain excellent real-time dynamic 3D shape reconstruction results, which may promote the development of the 3D shape reconstruction in SWIR band. Furthermore, SWIR 3D shape reconstruction can be pushed to a broader field, such as monitoring, by this work based on the characteristics of SWIR.

## Supplementary Materials

The following are available online at https://www.mdpi.com/1424-8220/20/2/521/s1, Video S1: 2D deformed fringe patterns versus real-time dynamic 3D shape reconstruction results.
